# Supplementary material for: Emergent metallicity at the grain boundaries of higher-order topological insulators
Source: Sci Rep. 2023 Sep 15;13:15308. doi: 10.1038/s41598-023-42279-8 (PMC10504356; doi:10.1038/s41598-023-42279-8)
Supplement: Supplementary file 1 — Supplementary Information. [file 41598_2023_42279_MOESM1_ESM.pdf]

Supplementary Information: Emergent metallicity at the grain boundaries of higher-order topological insulators

Daniel J. Salib,[1] Vladimir Juričić,[2,3] and Bitan Roy[1]
[1]Department of Physics, Lehigh University, Bethlehem, Pennsylvania, 18015, USA
[2]Departamento de Física, Universidad Técnica Federico Santa María, Casilla 110, Valparaíso, Chile
[3]Nordita, KTH Royal Institute of Technology and Stockholm University,
Hannes Alfvéns väg 12, SE-106 91 Stockholm, Sweden
(Dated: August 2, 2023)

In this Supplementary Information, we present (1) the explicit representation for the Γ matrices used for the numerical calculation and the symmetry analysis of higher-order topological insulators in both two and three dimensions [Sec. S1]. Additionally, we demonstrate (2) the stability of topological grain boundary modes against weak disorder [Sec. S2], and (3) the scaling of the gap between grain boundary and bulk modes with the amplitude of higher-order masses [Sec. S3].

S1. MATRIX REPRESENTATION AND SYMMETRY ANALYSIS

The Hamiltonian operator describing a second-order topological insulator in two dimensions takes the explicit form

$$\hat{h}^{\rm 2D}_{\rm SOTI} = t\left[\sin(k_x a)\Gamma_1 + \sin(k_y a)\Gamma_2\right] + \left(\Delta_1 - 2B\left[2 - \cos(k_x a) - \cos(k_y a)\right]\right)\Gamma_3 + \Delta_2\left\{\cos\theta\left[\cos(k_x a) - \cos(k_y a)\right] + \sin\theta\left[\sin(k_x a)\sin(k_y a)\right]\right\}\Gamma_4, \tag{S1}$$

where a is the lattice spacing. The mutually anticommuting four-component Hermitian Γ matrices are chosen to be (without any loss of generality)

$$\Gamma_1 = \sigma_3 \otimes \tau_1,\ \Gamma_2 = \sigma_3 \otimes \tau_2,\ \Gamma_3 = \sigma_3 \otimes \tau_3,\ \Gamma_4 = \sigma_1 \otimes \tau_0,\ \Gamma_5 = \sigma_2 \otimes \tau_0, \tag{S2}$$

each of which squares to identity. Two sets of Pauli matrices $\{\tau_\mu\}$ and $\{\sigma_\mu\}$ respectively operate on the orbital and spin indices, where $\mu = 0, 1, 2, 3$. Here we work with the representation of the Pauli matrices such that the ones for $\mu = 0, 1, 3$ are purely real, while the one with $\mu = 2$ is purely imaginary.

The unitary particle-hole symmetry (also known as the sublattice or the chiral symmetry) operator that fully anti-commutes with $\hat{h}^{\rm 2D}_{\rm SOTI}$ is generated by Γ_5 as $\{\hat{h}^{\rm 2D}_{\rm SOTI}, \Gamma_5\} = 0$. Besides the unitary particle-hole symmetry, $\hat{h}^{\rm 2D}_{\rm SOTI}$ also enjoys an anti-unitary particle hole symmetry (as known as the particle-hole symmetry), generated by $\Theta^{\rm 2D}_{\rm SOTI} = U\mathcal{K}$, where $\mathcal{K}$ is the complex conjugation and $U = \Gamma_1$, such that $\{\hat{h}^{\rm 2D}_{\rm SOTI}, \Theta^{\rm 2D}_{\rm SOTI}\} = 0$.

Notice that $\hat{h}^{\rm 2D}_{\rm SOTI}$ breaks the (1) time-reversal symmetry, generated by $\mathcal{T} = (\sigma_2 \otimes \tau_1)\mathcal{K}$, such that $\mathcal{T}^2 = -1$, (2) the four-fold rotational symmetry (C_4) about the z direction, generated by $R = \exp[i\pi(\sigma_0 \otimes \tau_3)/4]$ under which $\boldsymbol{k} = (k_x, k_y) \to (-k_y, k_x)$, and (3) parity (inversion) symmetry ($\mathcal{P}$), generated by Γ_3 and under which $\boldsymbol{k} \to -\boldsymbol{k}$. Thus, a 2D second-order topological insulator preserves the composite $C_4\mathcal{T}$, $C_4\mathcal{P}$ and $\mathcal{PT}$ symmetries. Notice that the $\mathcal{T}$, $\mathcal{P}$ and C_4 symmetries are separately broken only when Δ_2 is finite.

The Hamiltonian operator describing a second-order topological insulator in three dimensions takes the explicit form

$$\hat{h}^{\rm 3D}_{\rm SOTI} = t\left[\sin(k_x a)\Gamma_1 + \sin(k_y a)\Gamma_2 + \sin(k_z a)\Gamma_3\right] + \left(\Delta_1 - 2B\left[3 - \cos(k_x a) - \cos(k_y a) - \cos(k_z a)\right]\right)\Gamma_4 + \Delta_2\left\{\cos\theta\left[\cos(k_x a) - \cos(k_y a)\right] + \sin\theta\left[\sin(k_x a)\sin(k_y a)\right]\right\}\Gamma_5. \tag{S3}$$

In this case, the mutually anticommuting four-component Hermitian Γ matrices belong to the representation

$$\Gamma_1 = \tau_3 \otimes \sigma_1,\ \Gamma_2 = \tau_3 \otimes \sigma_2,\ \Gamma_3 = \tau_3 \otimes \sigma_3,\ \Gamma_4 = \tau_1 \otimes \sigma_0,\ \Gamma_5 = \tau_2 \otimes \sigma_0. \tag{S4}$$

As we have used all five mutually anticommuting Γ matrices in $\hat{h}^{\rm 3D}_{\rm SOTI}$, it does not possess any unitary particle-hole or sublattice or chiral symmetry. Still, an antiunitary operator $\Theta^{\rm 3D}_{\rm SOTI} = (\tau_2 \otimes \sigma_2)\mathcal{K}$ anticommutes with $\hat{h}^{\rm 3D}_{\rm SOTI}$ and

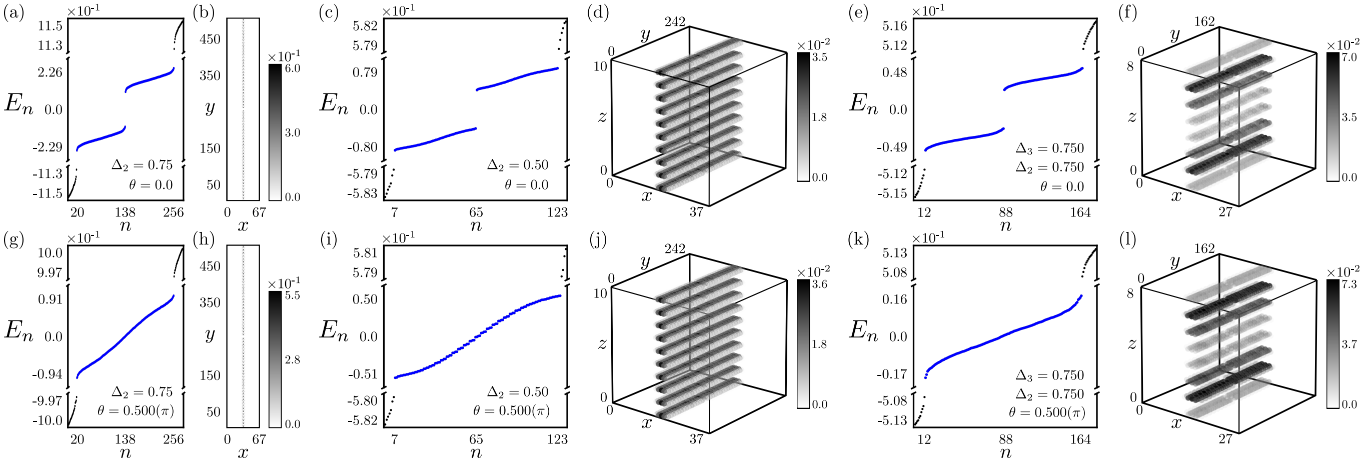

Figure S1. Stability of grain boundary (GB) modes against weak disorder. (a) Disorder-averaged energy spectrum of 2D second-order topological insulators hosting a grain-anti grain boundary pair with periodic boundary conditions in both directions. Defect (bulk) modes are shown in blue (black) for $\Delta_2 = 0.75$ and $\theta = 0$. (b) Corresponding local density of states of the GB modes [blue states in (a)]. (c) Similar to panel (a), but for a 3D second-order topological insulator with $\Delta_2 = 0.50$, $\theta = 0$, and periodic boundary condition in all three directions. (d) Corresponding local density of states for the GB modes [blue states in (c)]. (e) Similar to panel (a), but for a 3D third-order topological insulator with $\Delta_2 = \Delta_3 = 0.75$, $\theta = 0$, and open (periodic) boundary condition in the z (x and y) direction(s). (f) Corresponding local density of states of the GB modes [blue states in (e)]. Panels (g)-(l) are identical to panels (a)-(k), respectively, but for $\theta = \pi/2$. For 2D and 3D second-order topological insulators, all the details are identical to the ones reported in the captions of Figs. 2 and 3 of the main manuscript, respectively. For 3D third-order topological insulators this is also the case, except the GB and anti-GB defects contain 10 dislocations and anti-dislocations, respectively, and in the z direction the system size is $L_z = 8$. Throughout, we performed averaging over 24 random and independent disorder realizations. For more details consult Sec. S2.

generates its particle-hole symmetry.

Similar to its 2D counterpart, the 3D second-order topological insulator (only when Δ_2 is nonzero) also breaks the (1) time-reversal symmetry, generated by $\mathcal{T} = (\tau_0 \otimes \sigma_2)\mathcal{K}$, such that $\mathcal{T}^2 = -1$, (2) four-fold rotational symmetry about the z direction (C_4), generated by $R = \exp[i\pi(\tau_0 \otimes \sigma_3)/4]$, under which $\boldsymbol{k} = (k_x, k_y) \to (-k_y, k_x)$, and (3) parity (inversion) symmetry ($\mathcal{P}$), generated by Γ_4 and under which $\boldsymbol{k} \to -\boldsymbol{k}$. But, $\hat{h}^{\rm 3D}_{\rm SOTI}$ preserves the composite $C_4\mathcal{T}$, $C_4\mathcal{P}$ and $\mathcal{PT}$ symmetries.

In three dimensions, it is also conceivable to find a third-order topological insulator, for which the Hamiltonian operator reads as

$$\begin{aligned}\hat{h}^{\rm 3D}_{\rm TOTI} = t\left[\sin(k_x a)\Gamma_1 + \sin(k_y a)\Gamma_2 + \sin(k_z a)\Gamma_3\right] + \left(\Delta_1 - 2B\left[3 - \cos(k_x a) - \cos(k_y a) - \cos(k_z a)\right]\right)\Gamma_4 \\ + \Delta_2\left\{\cos\theta\left[\cos(k_x a) - \cos(k_y a)\right] + \sin\theta\left[\sin(k_x a)\sin(k_y a)\right]\right\}\Gamma_5 + \Delta_3\left[2\cos(k_z a) - \cos(k_x a) - \cos(k_y a)\right]\Gamma_6.\end{aligned} \tag{S5}$$

Since $\hat{h}^{\rm 3D}_{\rm TOTI}$ involves six mutually anti-commuting Hermitian matrices, their minimal dimensionality has to be eight. We choose the following representation

$$\begin{aligned}&\Gamma_1 = \eta_3 \otimes \tau_3 \otimes \sigma_1,\ \Gamma_2 = \eta_3 \otimes \tau_3 \otimes \sigma_2,\ \Gamma_3 = \eta_3 \otimes \tau_3 \otimes \sigma_3,\ \Gamma_4 = \eta_3 \otimes \tau_1 \otimes \sigma_0,\ \Gamma_5 = \eta_3 \otimes \tau_2 \otimes \sigma_0, \\ &\Gamma_6 = \eta_1 \otimes \tau_0 \otimes \sigma_0,\ \Gamma_7 = \eta_2 \otimes \tau_0 \otimes \sigma_0.\end{aligned} \tag{S6}$$

The newly introduced set of Pauli matrices $\{\eta_\mu\}$ operate on the sublattice degrees of freedom. Notice that $\{\hat{h}^{\rm 3D}_{\rm TOTI}, \Gamma_7\} = 0$. Therefore, Γ_7 generates a unitary particle-hole or sulattice or chiral symmetry of $\hat{h}^{\rm 3D}_{\rm TOTI}$.

The model Hamiltonian for the 3D third-order topological insulator breaks the (1) time-reversal symmetry, generated by $\mathcal{T} = (\eta_0 \otimes \tau_0 \otimes \sigma_2)\mathcal{K}$, such that $\mathcal{T}^2 = -1$, (2) four-fold rotational symmetry about the z direction (C_4), generated by $R = \exp[i\pi(\eta_0 \otimes \tau_0 \otimes \sigma_3)/4]$, under which $\boldsymbol{k} = (k_x, k_y) \to (-k_y, k_x)$, and (3) parity (inversion) symmetry

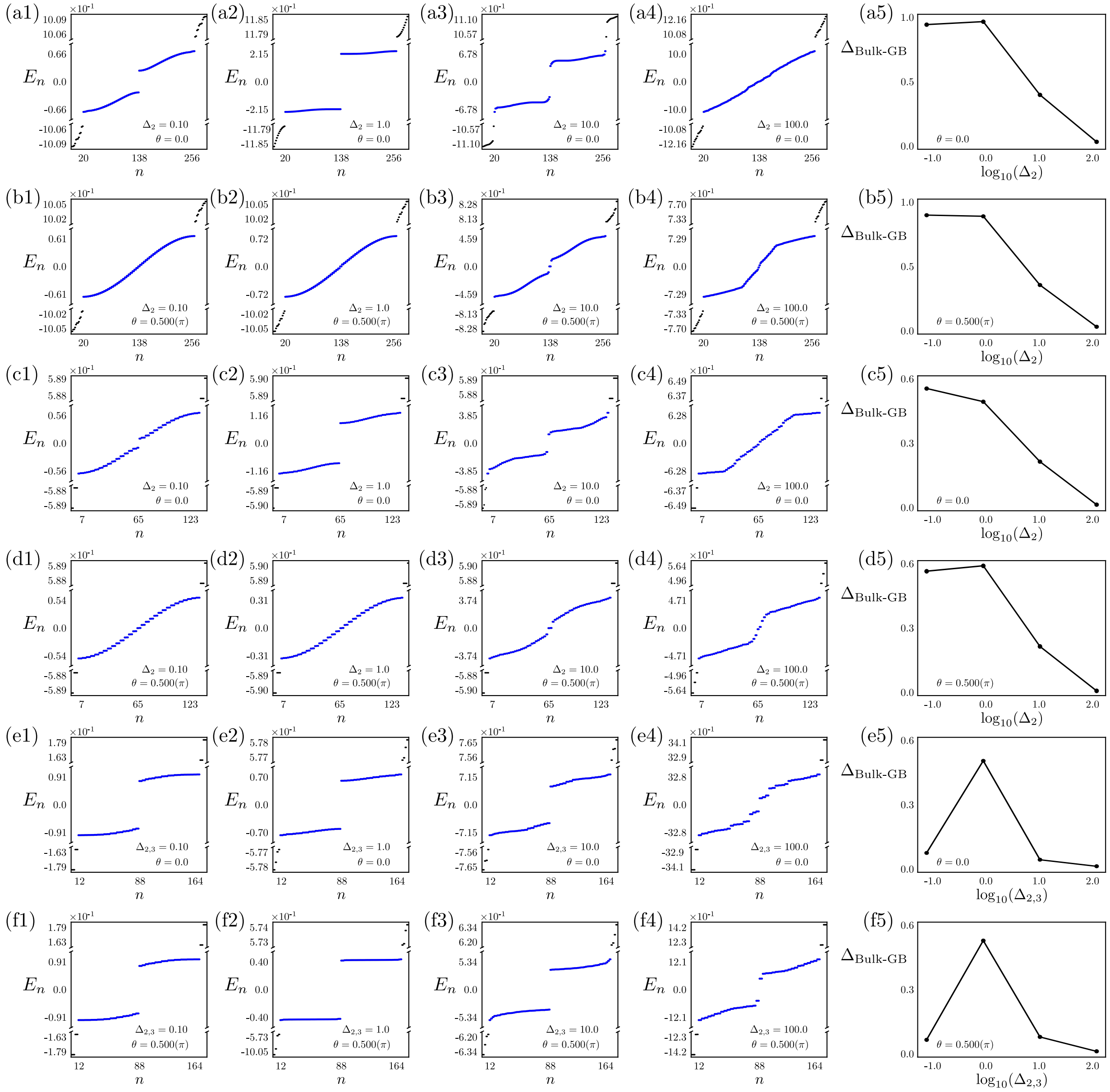

Figure S2. Energy spectra for 2D second-order topological insulators with $\theta = 0$ [(a1)-(a4)] and (b) $\theta = \pi/2$ [(b1)-(b4)], 3D second-order topological insulators with $\theta = 0$ [(c1)-(c4)] and (b) $\theta = \pi/2$ [(d1)-(d4)], 3D third-order topological insulators with $\theta = 0$ [(e1)-(e4)] and (b) $\theta = \pi/2$ [(f1)-(f4)] for various choices of the amplitude of the higher-order mass(es), the values of which are quoted in each panel. In the fifth column of each row, we show the scaling of the gap between the GB and bulk modes with $\log_{10}(\Delta_2)$ (for 2D and 3D second-order topological insulator) and $\log_{10}(\Delta_{2,3})$ with $\Delta_2 = \Delta_3$ (3D third-order topological insulator). The system size in each case is identical to the one reported in the caption of Fig. S1.

($\mathcal{P}$), generated by $\eta_0 \otimes \tau_1 \otimes \sigma_0$ and under which $\boldsymbol{k} \to -\boldsymbol{k}$. Thus, $\hat{h}^{\text{3D}}_{\text{TOTI}}$ preserves the composite $C_4\mathcal{T}$, $C_4\mathcal{P}$ and $\mathcal{PT}$ symmetries.

S2. STABILITY OF GRAIN BOUNDARY MODES AGAINST WEAK DISORDER

In this section, we discuss the stability of the grain-boundary modes against weak disorder. First, we numerically compute the bulk gap (G_{bulk}) for each of the higher-order topological insulators, namely 2D and 3D second-order

and 3D third-order topological insulators in a system with no lattice defects and with periodic boundary conditions in all directions, for specific value(s) of the higher-order mass(es). All other parameters are kept unchanged from the main manuscript. For a 2D second-order topological insulator we find $G_{\text{bulk}} = 2.4$ for $\Delta_2 = 0.75$ (in a system with $L_x = L_y = 22$), for 3D second-order topological insulator $G_{\text{bulk}} = 1.71$ for $\Delta_2 = 0.5$ (in a system with $L_x = L_y = L_z = 10$) and for 3D third-order topological insulator $G_{\text{bulk}} = 1.75$ for $\Delta_2 = \Delta_3 = 0.75$ (in a system with $L_x = L_y = L_z = 10$).

Random charge scatters are then introduced in the system with a GB-anti GB pair of defects by adding the following term to the real space Hamiltonian for higher-order topological insulators

$$H_{\text{dis}} = \sum_{\boldsymbol{r}_i} V(\boldsymbol{r}_i)\Gamma_0, \tag{S7}$$

where i is the site index, located at $\boldsymbol{r}_i$ and Γ_0 is four-dimensional (eight-dimensional) identity matrix for 2D and 3D second-order (3D third-order) topological insulator. Thus, $V(\boldsymbol{r}_i)$ represents a potential disorder, the dominant source of elastic scattering in any real material, distributed uniformly and independently within the range $[-W/2, W/2]$. Here, W denotes the strength of disorder, which we choose to be $W = G_{\text{bulk}}/10$, corresponding to *weak* disorder. For each realization of potential disorder $\text{Tr}[V(\boldsymbol{r_i})] = \Delta \neq 0$, and it is not particle-hole symmetric. Particle-hole symmetry is, however, recovered after averaging over a sufficiently large number of independent disorder realizations. To circumvent this issue, we define a quantity $\delta = \Delta/N$, where N is the total number of sites in the system, and subtract δ uniformly from all the sites. Naturally, $\delta \ll W$, and error introduced in this process is negligible. We perform the numerical simulations with a modified disorder Hamiltonian $H^{\text{mod}}_{\text{dis}} = H_{\text{dis}} - \sum_{\boldsymbol{r}_i} \delta\, \Gamma_0$. Notice that $H^{\text{mod}}_{\text{dis}}$ preserves the particle-hole symmetry for each disorder realization. For all the cases, we average over 24 independent disorder realization and compute (1) disorder-averaged energy spectra and (2) corresponding local density of states of the GB modes, which in the weak disorder regime remain well separated from the bulk states. The results are shown in Fig. S1. They establish the stability of GB modes for weak disorder.

S3. GAP BETWEEN BULK AND GRAIN BOUNDARY MODES WITH HIGHER-ORDER MASS

In this section, we discuss the scaling of the gap between the GB and bulk modes. For concreteness, we focus on the conduction band (for which all energy eigenvalues are strictly positive). Then we define a quantity $\Delta_{\text{bulk}-\text{GB}}$, measuring the energy gap between the largest eigenvalue for the GB modes and the smallest eigenvalue of the bulk states. Note that in the universal model Hamiltonian for higher-order topological insulators, there are first-order and higher-order masses. While the first-order mass remains finite throughout the 2D and 3D Brillouin zones (BZs), and therefore at all of the high symmetry points, the second-order Wilson-Dirac mass vanishes at the $\Gamma = (0, 0)$ and $\text{M} = (1, 1)\pi/a$ points of the 2D BZ. Similarly, in a 3D BZ, both the second-order and third-order Wilson-Dirac masses vanish at the $\Gamma = (0, 0, 0)$ and $\text{R} = (1, 1, 1)\pi/a$ points. As the accompanying Hermitian Γ matrices mutually anticommute with each other, the bulk gap is determined by the square-root of the sum of the squares of the first-order and higher-order masses. On the other hand, the energy scale of the GB modes is determined by the amplitude of the higher-order mass(es) and the parameter θ, which determines the domain-wall orientation of the discrete symmetry-breaking Wilson-Dirac mass. In the limit when the higher-order mass(es) is (are) sufficiently large, the energy scale of all the states is primarily determined by it. However, all the higher-order masses vanish at some of the high-symmetry points of the BZ. As a result, $\Delta_{\text{bulk}-\text{GB}}$ becomes extremely small. This gap is then solely determined by the energy of the first-order Dirac mass. Although the first-order Dirac mass is then sufficiently weaker than its higher-order counterpart(s), it is always finite in the entire BZ. Consequently, $\Delta_{\text{bulk}-\text{GB}}$ never disappears. Although, they may become hard to track in finite size systems, the GB modes can always be identified from energy and space resolved local density of states by scanning tunneling microscope measurements. These results are shown in Fig. S2.
